# Mapping the landscape of biliary tract cancer in Europe: challenges and controversies

**DOI:** 10.1016/j.lanepe.2024.101171

**Published:** 2025-02-19

**Authors:** Lorenza Rimassa, Shahid Khan, Bas Groot Koerkamp, Stephanie Roessler, Jesper B. Andersen, Chiara Raggi, Ana Lleo, Jean-Charles Nault, Julien Calderaro, Chiara Gabbi, Jakob N. Kather, Jesus M. Banales, Irene Bargellini, Helen Morement, Marcin Krawczyk, Paraskevi A. Farazi, Guido Carpino, Matias A. Avila, Anna Saborowski, Vincenzo Cardinale, Chiara Braconi, Rocio I.R. Macias

**Affiliations:** a Department of Biomedical Sciences, Humanitas University, Via Rita Levi Montalcini 4, 20072, Pieve Emanuele, Milan, Italy; b Medical Oncology and Hematology Unit, Humanitas Cancer Center, IRCCS Humanitas Research Hospital, Via A. Manzoni 56, 20089, Rozzano, Milan, Italy; c Department of Metabolism, Digestion and Reproduction, Imperial College London, Liver Unit, St Mary's Hospital Campus, South Wharf Road, W2 1NY, London, UK; d Department of Surgery, Erasmus MC Cancer Institute, Doctor Molewaterplein 40, 3015 GD, Rotterdam, the Netherlands; e Heidelberg University, Medical Faculty, Institute of Pathology, University Hospital Heidelberg, Im Neuenheimer Feld 224, 69120, Heidelberg, Germany; f Biotech Research & Innovation Centre (BRIC), Department of Health and Medical Sciences, University of Copenhagen, Ole Maaløes Vej 5, Copenhagen N, DK-2200, Denmark; g Department of Experimental and Clinical Medicine, University of Florence, Cubo Centro Polivalente 2, Viale Pieraccini 6, 50139, Florence, Italy; h Internal Medicine and Hepatology Unit, Department of Gastroenterology, IRCCS Humanitas Research Hospital, Via Manzoni 56, 20089, Rozzano, Milan, Italy; i Cordeliers Research Center, Sorbonne University, Inserm, Paris Cité University, “Functional Genomics of Solid Tumors” Team, Ligue Nationale Contre le Cancer Accredited Team, Labex OncoImmunology, 16 rue de l'École de Médecine, 75006, Paris, France; j Liver Unit, Avicenne Hospital, APHP, University Sorbonne Paris Nord, 125 Avenue de Stalingrad, 93000, Bobigny, France; k Université Paris Est Créteil, INSERM, IMRB, 61 Av. du Général de Gaulle, 94000, Créteil, France; l Department of Pathology, Assistance Publique-Hôpitaux de Paris, Henri Mondor-Albert Chenevier University Hospital, 1 Rue Gustave Eiffel, 94010, Créteil, France; m MINT-Hep, Mondor Integrative Hepatology, 1 Rue Gustave Eiffel, 94010, Créteil, France; n Humanitas Medical Care, Via Domodossola 9/a, 20145, Milan, Italy; o Else Kroener Fresenius Center for Digital Health, Faculty of Medicine and University Hospital Carl Gustav Carus, TUD Dresden University of Technology, 01307, Dresden, Germany; p Department of Medicine I, Faculty of Medicine and University Hospital Carl Gustav Carus, TUD Dresden University of Technology, 01307, Dresden, Germany; q Medical Oncology, National Center for Tumor Diseases (NCT), University Hospital Heidelberg, Im Neuenheimer Feld 224, 69120, Heidelberg, Germany; r Department of Liver and Gastrointestinal Diseases, Biogipuzkoa Health Research Institute – Donostia University Hospital, CIBERehd, Paseo Dr. Begiristain, s/n, 20014, San Sebastian, Spain; s IKERBASQUE, Basque Foundation for Science, Euskadi Pl., 5, Abando, 48009, Bilbao, Spain; t Department of Medicine, Faculty of Medicine and Nursing, University of the Basque Country (UPV/EHU), Barrio Sarriena, s/n, 48940, Leioa, Spain; u Department of Biochemistry and Genetics, School of Sciences, University of Navarra, Calle Irunlarrea 1, 31008, Pamplona, Spain; v Department of Surgical Sciences, University of Turin, Corso Dogliotti 14, 10126, Turin, Italy; w Division of Diagnostic and Interventional Radiology, Candiolo Cancer Institute FPO-IRCCS, Strada Provinciale 142, 10060, Candiolo (TO), Italy; x AMMF – The Cholangiocarcinoma Charity, Enterprise House, Bassingbourn Road, Stansted, CM24 1QW, Essex, UK; y Department of Gastroenterology, Hepatology and Transplant Medicine, Medical Faculty, University of Duisburg-Essen, Hufelandstraße 55, 45147, Essen, Germany; z Laboratory of Metabolic Liver Diseases, Medical University of Warsaw, Banacha Street 1B, 02-097, Warsaw, Poland; aa School of Medicine, European University Cyprus, 6 Diogenes Street, 2404, Engomi, Nicosia, Cyprus; ab Department of Anatomical, Histological, Legal Medicine and Orthopedic Sciences, Sapienza University of Rome, Via Alfonso Borelli 50, 00161, Rome, Italy; ac Hepatology Laboratory, Solid Tumors Program, CIMA, IdiSNA, CIBERehd, University of Navarra, Calle Irunlarrea 1, 31008, Pamplona, Spain; ad Department of Gastroenterology, Hepatology, Infectious Diseases and Endocrinology, Hannover Medical School, Carl Neuberg Str. 1, 30625, Hannover, Germany; ae Department of Translational and Precision Medicine, Sapienza University of Rome, Via Alfonso Borelli 50, 00161, Rome, Italy; af School of Cancer Sciences, University of Glasgow, Switchback rd, G61 1QH, Glasgow, UK; ag Beatson West of Scotland Cancer Centre, 1053 Great Western rd, G12 0YN, Glasgow, UK; ah CRUK Scotland Cancer Centre, G61 1BD, Glasgow, UK; ai Experimental Hepatology and Drug Targeting (HEVEPHARM) Group, University of Salamanca, IBSAL, CIBERehd, Campus M. Unamuno s/n, 37007, Salamanca, Spain

**Keywords:** Biliary tract cancer, Cholangiocarcinoma, Gallbladder carcinoma, Epidemiology, Europe, Access to diagnosis, Biomarkers

## Abstract

Biliary tract cancer (BTC) is becoming more common worldwide, with geographic differences in incidence and risk factors. In Europe, BTC may be associated with primary sclerosing cholangitis, lithiasis, and liver cirrhosis, but is more frequently observed as a sporadic disease. BTC increasingly affects patients under 60 years, resulting in a significant social and economic burden. Early diagnosis remains challenging due to vague symptoms in 50% of patients with BTC, and lack of specific biomarkers, resulting in late presentation and poor prognosis. The identification of patients at increased risk and reliable biomarkers require collaborative efforts to make faster progress. This Series paper highlights the disparities in access to diagnostic tools and multidisciplinary care in Europe, particularly in economically disadvantaged regions, while identifying priority areas for improvement. Addressing these inequities requires harmonised guidelines, accelerated pathways to curative treatments, and improved awareness among healthcare professionals and the public. Multidisciplinary teams (MDTs) are crucial for the diagnosis of BTC and for improving patient outcomes, yet inconsistencies exist in their implementation not only between different countries, but also between different centres within a country. Collaboration and standardisation of diagnostic and treatment protocols across Europe are essential to effectively address the management of patients with BTC.

## Introduction

Biliary tract cancer (BTC) refers to a group of heterogenous malignancies arising within the epithelium of the bile ducts. BTC include gallbladder cancer (GBC) and cholangiocarcinoma (CCA) in its three subtypes depending on the segments of bile duct involved [intrahepatic (iCCA), perihilar (pCCA) and common bile duct distal to the cystic duct (dCCA)]. Of note, from a clinical point of view, pCCA and dCCA have been frequently grouped as extrahepatic CCA (eCCA) in the past with implications on interpretations of historical data.

**Key messages**

▪ A diagnostic pathway for patients with symptoms suggestive of BTC should be established and shared across secondary care physicians, who should be aware of this cancer to allow prompt and effective referral, as should emergency departments and secondary care centres, to make sure that diagnosis is not delayed.
▪ All patients with BTC should be discussed within an MDT, correctly classified and offered a multidisciplinary management plan. This would support also a correct registration of patients for epidemiological studies and clinical registries.
▪ A correct ICD-coding should be recorded for each patient with BTC in each institution to reflect real epidemiological trends. Current data suggests that the incidence of iCCA is increasing globally with differences across European countries that need to be analysed.
▪ Further research is needed for the development of early diagnostic biomarkers, and this should be supported by appropriate and dedicated funding streams.

**Search strategy and selection criteria**

References for this Series paper were identified through searches of PubMed with the search terms “biliary cancers”, “epidemiology”, “MDT” from 2010 until 2024. Articles were also identified through searches of the authors’ own files. Only papers published in English were reviewed. The final reference list was generated on the basis of originality and relevance to the broad scope of this Series paper.

Incidence of BTC is increasing worldwide.^1^^,^^2^ While in East Asia, CCA is primarily associated to infestations with *Clonorchis sinensis* and *Opistorchis viverrini* (liver flukes) and GBC with *Salmonella thiphy* infection, in Europe it is related to primary sclerosing cholangitis (PSC), lithiasis and other risk factors associated with the development of liver cirrhosis.^1^^,^^3^

Age of onset of BTC has reduced over the years, with 30% of patients being younger than 60 years at present times,^4^^,^^5^ thus affecting the working population and generating a relevant socio-economic burden on the society. Sex differences and ethnic/racial factors also play a key role. Women are affected by GBC up to three times more than men,^6^ while the incidence of iCCA is higher in males, particularly in Hispanics.^7^

Early diagnosis continues to represent a major issue due to the presence of vague symptoms and the lack of specific diagnostic biomarkers. Diagnosis usually comes late via the Emergency Department, with 50% of patients presenting with acute symptoms,^5^^,^^8^ after 12–18 months from the initial symptomatology.^9^

Involvement of several medical professionals is essential for the diagnosis and management of patients with BTC, and discussions at MultiDisciplinary Team (MDT) meetings should be favoured to optimise the patients’ journey through to the treatment. However, implementation of MDT discussion is not fully harmonised amongst different institutions and countries across Europe.^10^ The factors discussed above, along with the lack of awareness of BTC, raise the need to not only disseminate international guidelines, but assess the adherence across European countries and discuss a plan for harmonisation of the management of patients with BTC at an international level. Here we will discuss a multidisciplinary viewpoint on the diagnosis of BTC, highlighting inequities around Europe and identifying priority areas for improvement.

## Epidemiology in Europe

**Fig. 1:**
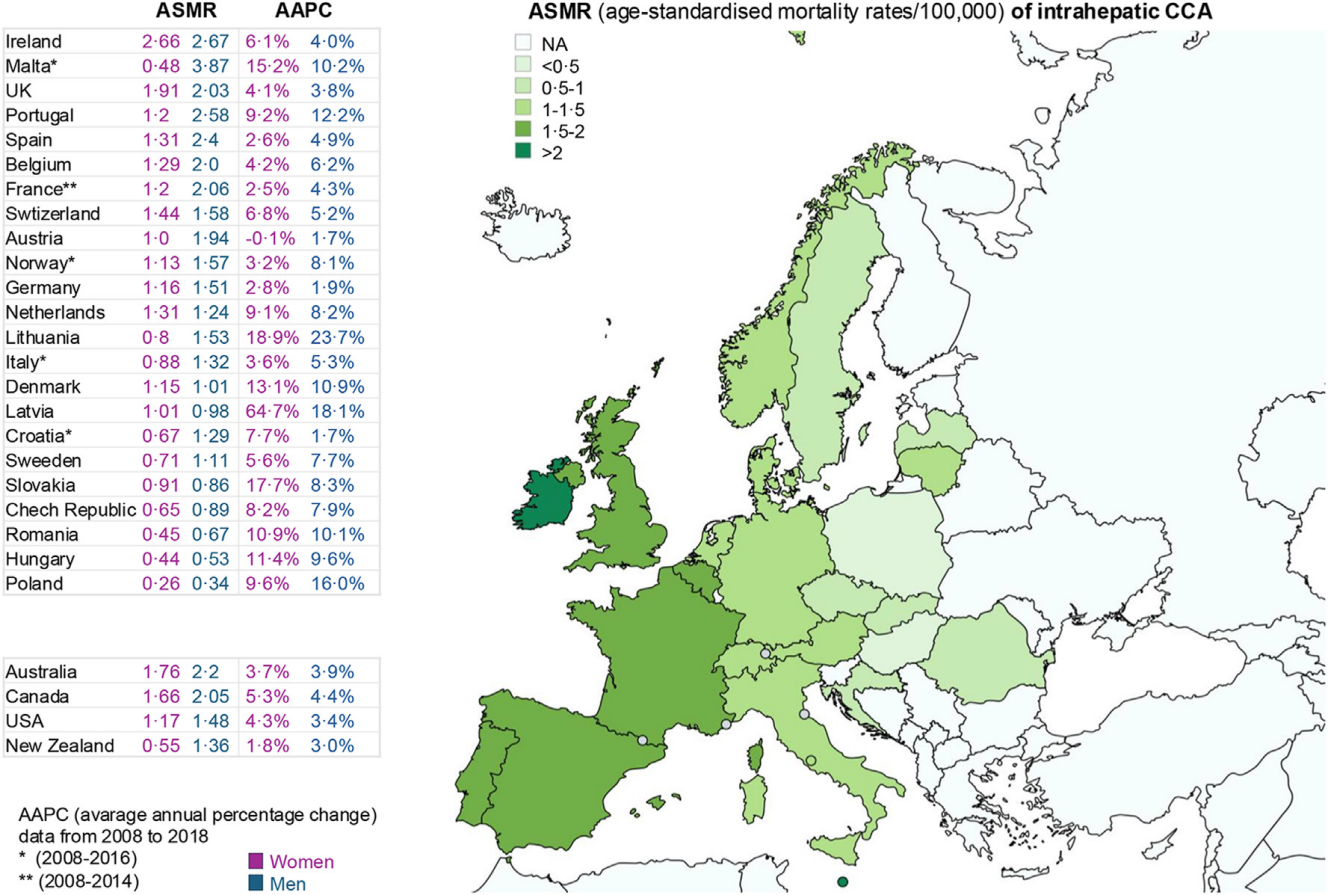
**Worldwide mortality (age-standardised mortality rates [ASMR]/100,000) of intrahepatic CCA and average annual percentage change (AAPC) in 23 European countries compared with the USA, Canada, Australia and New Zealand (data extracted with permission from**^15^**)**. ASMR in European countries are ordered from highest to lowest considering the average between men and women. NA, not available.

**Fig. 2:**
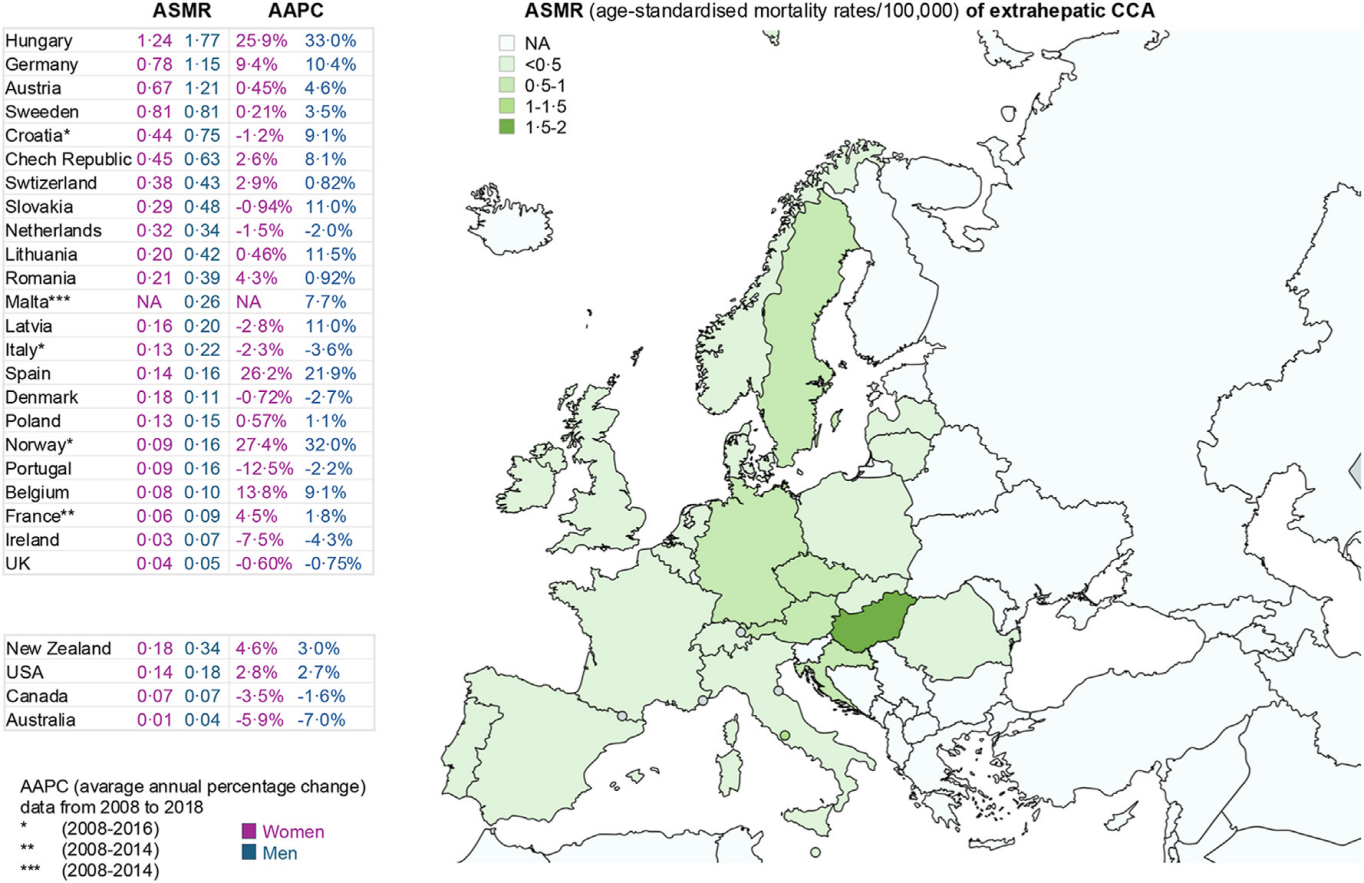
**Worldwide mortality (age-standardised mortality rates [ASMR]/100,000) of extrahepatic CCA and average annual percentage change (AAPC) in 23 European countries compared with the USA, Canada, Australia and New Zealand (data extracted with permission from**^15^**).** ASMR in European countries are ordered from highest to lowest considering the average between men and women. NA, not available.

CCA incidence exhibits geographical variation, with much higher incidence in parts of the Eastern world compared to the West, reflecting geographical differences in risk factors, both genetic and environmental.^11^^,^^12^ Over recent decades, the incidence rates of CCA subtypes showed distinct trends: iCCA rising and eCCA stable/decreasing.^1^^,^^11^^,^^12^ Recent observations in the United States (US) (period 2001–2017) provide further evidence for an increase in iCCA incidence in Western countries (148.8%; 0.80–1.99 per 100,000 person-years) and confirmed a stable incidence of pCCA/dCCA (7.5%; 2.28–2.45).^13^ Notably, the greatest increase was among younger patients (18–44 years, 81.0%).^13^ Reflecting the incidence trends both, globally in Europe,^14^^,^^15^ and in Western countries,^15^ deaths due to iCCA (Fig. 1) are rising at a higher rate than deaths due to eCCA (Fig. 2), which levelled off or decreased. In Europe, Latvia showed the highest average annual percentage change in mortality rates of iCCA from 2008 to 2018, followed by Lithuania, Slovakia, Malta and Denmark. Notably, mortality rates are almost 40% higher in the most socioeconomically deprived areas.^5^ Rates of pCCA specifically are unknown due to the historic lack of a unique ICD code for it until the latest versions of ICD-11 and ICD-O-3.2, but pCCA may have previously been misassigned to iCCA rates.^16^

Various risk factors have been associated with iCCA and/or eCCA (Table 1).^42^ These include inflammatory biliary diseases that affect large intra-and/or extrahepatic bile ducts such as PSC (the commonest known predisposing cause of CCA in the West), secondary biliary cirrhosis, choledocholithiasis, hepatolithiasis, and cholecystitis (Table 1). These risk factors are infrequent but associated with a high CCA risk, primarily pCCA/dCCA. Liver flukes increase dramatically the risk of CCA. Other risk factors for CCA include obesity, smoking, alcohol intake of >80 g/day, metabolic syndrome including type 2 diabetes, alcohol-related disorders, chronic viral hepatitis B and C, and cirrhosis. Finally, occupational exposure to asbestos has been shown to increase the risk of both iCCA and pCCA/dCCA.^43^^,^^44^ Few studies have included European populations and have shown associations of inflammatory bowel disease^31^^,^^32^ and PSC^31^ with both iCCA and pCCA/dCCA, diabetes and liver cirrhosis with iCCA, and cholelithiasis and viral hepatitis C with pCCA/dCCA.^31^ Less data are available when two or more risk factors co-occur in the same patient. Despite advancements in our understanding of CCA aetiology, in Western countries at least 50% of cases are still diagnosed without any identifiable risk factor. While the increased incidence in metabolic disorders like obesity could partially explain the raise in CCA incidence, more studies are needed to understand if unknown risk factors could be responsible of the raising burden or more cases are now recognized as iCCA,^45^ rather than as cancer of unknown primary or hepatocellular carcinoma (HCC).

**Table 1:** Summary of risk factors significantly associated to intrahepatic CCA and/or extrahepatic CCA (eCCA) as assessed by case control studies (Odd Ratios by multivariate analyses).

Risk factors for iCCA (Ref)	Odds ratios for increased risk	Risk factors for eCCA (Ref)	Odds ratios for increased risk
**Bile duct diseases and conditions**		**Bile duct diseases and conditions**	
Cholecystitis^17^	8.5	Cholecystitis^17^	5.9
Caroli disease^18^	38.13	Caroli disease^18^	96.81
Cholelithiasis^18^^,^^19^	1.8–13.5	Cholelithiasis^17^, ^18^, ^19^, ^20^, ^21^	2.6–11
Hepatolithiasis^22^, ^23^, ^24^, ^25^,^b^	50.0–4.8; 6.7^b^	Hepatolithiasis^19^^,^^21^^,^^26^	3.09, 16.47
Choledochal cysts^17^, ^18^, ^19^^,^^24^^,^^27^^,^^28^	10.7–43.03	Choledochal cysts^17^, ^18^, ^19^	47.1, 27.12
Choledocholithiasis^18^^,^^20^^,^^25^	4.17–33.35, 6.94	Choledocholithiasis^17^^,^^18^^,^^21^^,^^26^^,^^29^	6.68–34
Cholangitis/PSC^17^^,^^19^^,^^27^	64.2–75.23, 93.4	Cholangitis/PSC^17^^,^^19^	45.7, 453 (pCCA), 34 (dCCA)
Non-PSC related cirrhosis^19^	13.8	Non-PSC related cirrhosis^19^	14.1 (pCCA)
Biliary cirrhosis/PBC^17^^,^^18^^,^^27^	17.08–19.8, 9.84	Biliary cirrhosis/PBC^17^^,^^18^	11.8, 8.34
Cholecystectomy^17^^,^^18^^,^^30^	2.74–5.4	Cholecystectomy^17^^,^^18^^,^^20^^,^^21^	4.71–12
**Digestive diseases**		**Digestive diseases**	
Inflammatory bowel disease^17^^,^^31^^,^^32^	1.72–3.95	Inflammatory bowel disease^17^^,^^32^	1.1, 1.97
Crohn's disease^17^^,^^18^^,^^27^	1.68–2.4	Crohn's disease^17^^,^^18^	2.8, 1.71
Gout^18^	1.4	Gout^18^	1.43
Thyrotoxicosis^18^	1.25		
Ulcerative colitis^17^^,^^18^^,^^27^	2.18–4.5,	Ulcerative colitis^18^	1.75
Duodenal ulcer^17^^,^^18^	3.4, 1.42	Duodenal ulcer^17^^,^^18^	1.9, 1.46
Chronic pancreatitis^17^^,^^18^	5.9, 2.66	Chronic pancreatitis^17^^,^^18^	9.3, 6.61
**Liver flukes**		**Liver flukes**	
*Clonorchis sinensis* infection^33^^,^^34^	8.6–13.6	*Clonorchis sinensis* infection^34^	6.5
*Opistorchis felineus* infection^35^	3.9	*Opistorchis felineus* infection^35^	3.9
**Endocrine disorders**		**Endocrine disorders**	
Thyrotoxicosis^17^	1.5	Thyrotoxicosis^17^	1.7
Type II diabetes^18^^,^^19^^,^^24^^,^^25^^,^^33^^,^^36^^,^^37^	1.8–3.6	Type II diabetes^17^, ^18^, ^19^, ^20^, ^21^^,^^26^	1.5–3.2, 3.36, 1.45; 2.88 (pCCA), 4.22 (dCCA)
Type I diabetes^18^	1.43	Type 1 diabetes^18^	1.3
**Metabolic conditions and general risks**		**Metabolic conditions and general risks**	
Obesity^17^^,^^18^^,^^27^	1.42–1.71,	Obesity^18^	1.17
Alcohol intake >80 g/day^24^^,^^30^^,^^36^	1.52–5.21	Alcohol intake >80 g/day^21^^,^^29^^,^^30^	1.05–3.6,
Alcohol-related disorders^18^	3.72	Alcohol-related disorders^18^	2.6
Smoking^17^, ^18^, ^19^^,^^27^^,^^37^	1.3–2.1	Smoking^17^, ^18^, ^19^^,^^29^^,^^38^	1.25–2.52 (pCCA), 1.85 (dCCA)
Metabolic syndrome (overall)a,^28^	1.32–1.83		3.15
Dyslipidemia^18^	1.41	Dyslipidemia^18^	1.56
Dyslipoproteinemia^27^	1.65		
Hypertension^27^	1.63, 1.39	Hypertension^18^	1.43
		Family history of other cancer^21^	3.15
**Chronic liver diseases**		**Chronic liver diseases**	
Alcoholic liver disease^17^^,^^27^	3.1–5.69	Alcoholic liver disease^17^	4.5
Non-specific cirrhosis^17^, ^18^, ^19^, ^20^, ^21^, ^22^, ^23^, ^24^, ^25^, ^26^, ^27^, ^28^, ^29^, ^30^, ^31^^,^^36^^,^^37^	8.26–28.79,	Non-specific cirrhosis^17^, ^18^, ^19^, ^20^, ^21^, ^22^, ^23^, ^24^, ^25^, ^26^, ^27^, ^28^, ^29^, ^30^, ^31^	1.6–6.16
Hemochromatosis^17^^,^^18^	2.6, 2.07		
Hepatic schistosomiasis^25^	11		
Non-alcoholic liver disease^17^^,^^18^	3, 3.52	Non-alcoholic liver disease^18^	2.93
Unspecified viral hepatitis^27^	7.66		
HCV infectionb,^18^^,^^19^^,^^23^, ^24^, ^25^^,^^31^^,^^28^^,^^34^^,^^37^	2.41–9.71; 9.7^b^	HCV infection^18^^,^^19^^,^^39^	1–3.05, 3.18, 3.51 (pCCA)
HCV infection plus cirrhosis^22^	8.53		
HBsAg positivec,^19^^,^^20^^,^^25^^,^^31^^,^^28^^,^^34^^,^^37^^,^^41^	2.3–12.9 2.35–4.3^c^	HBsAg positivec,^19^^,^^32^^,^^22^^,^^41^	1.84–16.8 0.92–2.14^c^
HBsAg positive plus cirrhosis^20^^,^^22^^,^^38^	13–18	HBsAg positive plus cirrhosis^21^	3.42
HBsAg negative/HBcAb positivec,^22^^,^^41^	1.09–1.81^c^	HBsAg negative/HBcAb positive^c^^,^^21^^,^^40^	1.50 0.88–1.24^c^
		**AB0 blood types**	
		AB0 blood type A^21^	1.78
		AB0 blood type B^21^	1.27
		AB0 blood type AB^21^	0.44
		AB0 blood type and HbsAg positive^21^	3.04
		AB0 blood type A and HbsAg positive/HBcAb positive^21^	3.79
**Occupational exposure**		**Occupational exposure**	
To asbestos^41^	4.81	To asbestos^41^	2.09

eCCA, extrahepatic cholangiocarcinoma (includes distal [dCCA] and perihilar cholangiocarcinoma [pCCA], the later are histological/cytological verified cases); HCV, hepatitis C virus; iCCA, intrahepatic cholangiocarcinoma (histological verified cases); PSC, primary sclerosing cholangitis.

a According the 2001 U.S. NCEP-ATP III definition.

b iCCA cases include 2 cases of cHCC-CCA.

c Risk of CCA only in Asia. Table updated from.^42^

For GBC, the lifetime risk of developing this type of cancer in 2020, considering both genders, was higher in Eastern European countries than in Western European countries.^46^ In particular, the highest incidence and age standardised mortality rates were reported in Poland, Hungary, Czech Republic, and Slovakia.^6^^,^^47^ Differences in metabolism and sex hormones may account for gender disparities in GBC and the use of hormonal replacement therapy in post-menopausal women has been described to further increase its risk.^48^ Other risk factors include metabolic alterations such as obesity and cholesterol gallstone disease, advanced age, as well as a status of chronic inflammation induced by recurrent gallbladder infections.^49^ Studies are needed to understand more precisely the epidemiological characteristics of each type of BTC in Europe.

## Diagnosis, biology and prognosis

BTCs are often diagnosed at an advanced stage due to their asymptomatic nature, leading to a poor prognosis.^1^^,^^50^ Complete (R0) resection significantly improves patient outcome and differences in prognosis among BTC subtypes can be partially explained by surgical opportunities.^2^^,^^51^ Separate diagnostic paths are required for each BTC subtype. For diagnosis and staging of CCA, integration of different imaging modalities, including transabdominal ultrasound, computed tomography (CT) and magnetic resonance imaging (MRI) are recommended depending on the clinical features. However, histology or cytology confirmation is recommended for definitive diagnosis for treatment stratification. In cases amenable to surgery, attempts to tissue diagnosis should be discussed and agreed at MDT before being implemented. In unresectable BTC, tissue biopsy of primary or metastatic lesions should be pursued before starting non-curative treatments. Liver tumour biopsy is most frequently performed in iCCA, GBC and BTC with liver metastases,^52^ while endoscopic ultrasonography-guided fine needle aspiration (EUS-FNA) alone or in combination with endoscopic retrograde cholangiopancreatography (ERCP) enables assessment of locoregional extension of pCCA, dCCA or GBC together with the pathologic evaluation.^53^^,^^54^ Brush cytology to discriminate eCCA from high-grade dysplasia remains challenging with a sensitivity <80%.^52^ When cytology or histology are inconclusive, a full discussion with the MDT could be useful for treatment selection.

For iCCA, differential diagnosis with liver metastases from other primary tumours is crucial, underpinning the requirement of tissue analysis. However, there is no specific immunohistochemical profile for distinguishing between iCCA and liver metastasis from upper gastrointestinal, pancreatic or extrahepatic biliary tumours. Therefore, routine immunohistochemical panels are not recommended, which could spare the biopsy material for molecular analysis.^55^^,^^56^ Deep learning advances utilizing H&E-stained slides to discriminate iCCA versus colorectal cancer liver metastasis or DNA methylation-based classifiers differentiating iCCA from intrahepatic pancreatic cancer may advance the field in the future.^57^^,^^58^ However, it will be crucial to assess how they perform to exclude adenocarcinomas of other origins. Besides, iCCA is further classified into small-duct and large-duct iCCA, which is currently only possible by histopathological evaluation.^52^^,^^59^ Small-duct and large-duct iCCA differ in prognosis, with patients with small-duct iCCA having overall better outcome.^59^^,^^60^ This might also be reflected by the higher prevalence of *IDH1/IDH2* mutations and *FGFR2* gene fusions in small-duct iCCA.^61^^,^^62^ In addition, small-duct and large-duct iCCA have distinct cells-of-origin and differ in their biology.^1^

As mentioned, in Europe, majority of patients with BTC have no known risk factors, which explains the high number of late-stage diagnosis.^14^^,^^63^ Still, surveillance programs for high-risk patients such as those with PSC, liver cirrhosis or viral hepatitis are warranted for early diagnosis. Current programs applying annual screening of serum carbohydrate antigen 19–9 (CA19-9) and contrast-enhanced MRI with cholangiopancreatography (MRI/MRCP) failed to improve long-term survival.^64^ This might be due to high CA19-9 levels in patients with severe bile duct changes, especially with underlying PSC. Therefore, novel diagnostic markers are urgently required. One recent study identified serum markers for prediction of CCA development before clinical evidence of malignancy and another study identified DNA methylation biomarkers in bile for early and accurate diagnosis of CCA in patients with PSC.^65^^,^^66^ Further studies are required for independent validation and development of clinical algorithms for patients with respective risk factors. Overall, recommendations for surveillance programmes for CCA onset in patients at risk are not fully supported by strong evidence, increasing the heterogeneity of clinical managment across Europe.

## Availability and accessibility of MDT in Europe

MDTs in oncology refer to collaborative groups of healthcare professionals from various specialties working together to provide comprehensive care tailored to each patient. An effective MDT integrates diverse clinical perspectives, allowing for coordinated and holistic treatment approaches to address the complex needs associated with poor prognosis cancers. These teams aim to improve patient outcomes by fostering open communication, shared decision-making and a patient-centred approach that takes into account not only the medical, but also the psychological, social and support needs of people facing difficult diagnoses. This collaborative model is crucial in ensuring a comprehensive and effective patient care. Indeed, a systematic review has shown that MDTs often result in improved compliance with guidelines, superior diagnostic accuracy, and increased adherence to treatment strategies of gastrointestinal malignancies.^67^ Such benefits have been demonstrated by numerous studies also for patients with BTC. Therefore, the European Society for Medical Oncology (ESMO) guideline recommends the standard inclusion of patients with BTC in MDT discussion,^68^ and a growing number of countries are including MDTs in national strategies to fight against cancer. For instance, well-structured and regular MDTs, as well as the discussion of all patients in MDT meetings, are listed among systemic tumour-specific quality indicators in multiple cancer society certification programs for multidisciplinary cancer centres.^69^ In addition, given the relevant advancements in the molecular diagnostics of patients with biliary tumours, recent national guidelines recommend the inclusion of patients with CCA in molecular tumour boards (MTBs) that encompass also the expertise of basic/medical scientists like molecular biologists and geneticists.^70^^,^^71^ Nevertheless, as recognised by international societies, access to molecular diagnostics in oncology is insufficient in many European countries. For instance, advanced biomolecular technologies remain largely inaccessible in clinical practice, with techniques such as whole exome sequencing (WES), RNA sequencing (RNA-Seq), and genomic assays predominantly available only in clinical trials or research settings within high-income countries, and rarely accessible in low- and middle-income countries. This shortfall significantly diminishes the likelihood of incorporating such data into MDT discussions.^72^ Evidence also highlights regional disparities in healthcare access and management of patients with CCA due to geographic and socioeconomic determinants, which could impact oncological outcomes.^73^^,^^74^ However, most of these studies have been conducted in the US) and limited data are available from Europe, where health systems are more heterogeneous,^75^ and sharing/comparing molecular details is complicated due to ethical and GDPR issues. Moreover, several studies suggest that patients treated for CCA at high-volume centres more frequently receive curative surgical treatments, with enhanced overall survival.^76^^,^^77^ Although these studies face selection biases and the exact threshold for defining high-volume centres is still debated, the data indicate that specialised centres, which possess technical expertise and advanced imaging access, with essential diagnostic tools like MRI, radiological, and pathological skills, and MDTs should manage patients with these rare cancers.

**Fig. 3:**
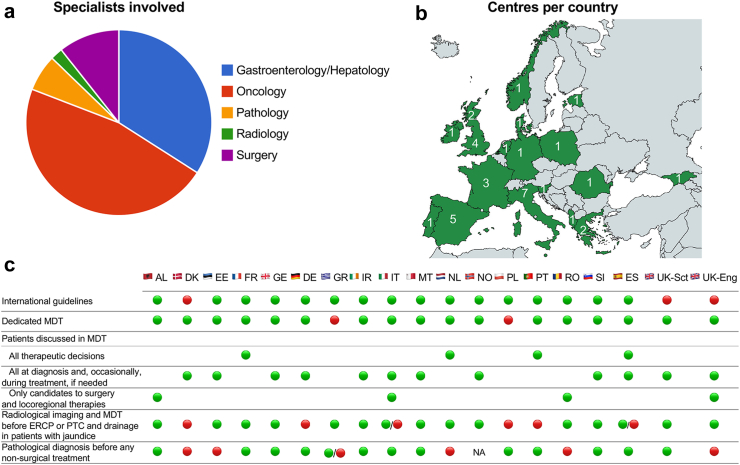
**Survey data from 47 clinicians from 36 European hospitals.** (a) Percentage of specialists involved in the survey. (b) Number of centres participating per country. (c) Management of patients with biliary tract cancer in a selection of European hospital in 19 countries. ERCP, endoscopic retrograde cholangiopancreatography; MDT, multidisciplinary team; PTC, percutaneous transhepatic cholangiography. , Yes; , No.

The analysis of data collected through a survey (Supplementary Table S1) involving 47 clinicians from 36 centres in 18 European countries supports differences in the patient management for BTC diagnosis (Fig. 3). Although the results must be interpreted with caution, there is an overall trend to apply international guidelines, even though implementation may be heterogenous depending on access to technologies and drugs. Some countries, such as Denmark, Italy and the United Kingdom (UK) have developed national guidelines to harmonise international recommendations to resources available in the country. Overall, more than 90% of the Institutions which participated in the survey, have an established MDT, with a variable degree of BTC-dedicated oncologists and radiologists. There is higher heterogeneity in the implementation of MDT discussion, with some institutions using the MDT as a formal registration/discussion for all patients at diagnosis to guide the multidisciplinary patients’ plan, while others discussing only selected cases where a different expertise is required from the managing physician (Fig. 3). It should be noted that the number of centres analysed is small and may not fully reflect the situation and heterogeneity in each country, since disparities exist not only across European nations but also within a single country. In Italy, for example, care pathways are defined partly at the national level and partly at the regional level, creating differences among the various regions. Dedicated MDTs, regional MTBs, access to and reimbursement for molecular profiling can vary across regions, which can lead to disparities in management.

The implementation of MTBs including molecular biologists and geneticists, together with the development of national research and clinical networks, will enhance the effective care of patients with BTC.

## Challenges

### Need to increase awareness

Low awareness amongst healthcare professionals (HCPs), health authorities, and the public, poses a major challenge, which needs to be addressed from a holistic approach, and is one where patient advocacy groups and collaborative initiatives with multi-stake holders, such as COST (European Cooperation in Science and Technology) Actions, can play a key role.

The main challenges in raising awareness are: i) the comparative rarity and complexity of CCA, and BTC in general, leading to a lack of familiarity among the public and even within the medical community. Symptoms are often nonspecific, easily attributed to more common conditions, leading to delayed diagnoses, ii) limited research and funding until recently; BTC receives less attention and funding compared to more prevalent cancers. This scarcity of resources hampers the development of effective treatments and early diagnostic tools. In addition, the BTC's complex biology requires specialised research, further complicating funding efforts, and iii) geographical disparities: awareness and healthcare infrastructures vary considerably across regions. In low-resource settings, lack of awareness and limited access to advanced medical care aggravate the problems faced by patients with CCA and other BTCs, underscoring the need for a global approach to awareness and advocacy.

Patient advocacy groups have a pivotal role to play. In the UK, the AMMF—The Cholangiocarcinoma Charity, the world's first CCA charity, plays a crucial role in raising awareness across the UK and Europe and in funding CCA research. Commissioning a first of its kind CCA data project in England,^5^^,^^8^^,^^78^ organising an annual international conference, distributing educational materials for patients and HCPs, providing discussion platforms for patients and leveraging social media, AMMF reaches a global audience. This is powerful in attracting the attention of the public, HCPs and policy makers. Collaborating with other CCA patient advocacy groups -the American Cholangiocarcinoma Foundation, the Italian APIC, the Spanish ATUVIBI and the Thai CCA Foundation-further enhances these efforts. Other associations are doing great work, such as the French ACABI. However, it should be noted that most countries do not have patient advocacy groups fully dedicated to BTC/CCA.

COST Action initiatives developed by the European Network for the Study of Cholangiocarcinoma (ENSCCA), such as Precision-BTC-Network (https://www.cost.eu/actions/CA22125/; https://precision-btc.eu) and previously EURO-CHOLANGIO-NET, are instrumental in fostering international collaboration. By bringing together multidisciplinary groups of researchers, clinicians, and international patient representatives, these COST Actions facilitate the exchange of knowledge, best practices and research findings, as well as of unified strategies to tackle BTC. These collaborations help standardise diagnostic and treatment protocols, thus improving patient outcomes globally.

Conducting educational campaigns targeting both the general public and medical professionals is essential. Workshops, webinars, and informational sessions can enhance understanding of BTC, its risk factors, and the importance of early detection. Engaging the media to highlight patient stories and advancements in research can further increase public interest and support.

### Need to increase early diagnoses

pCCA and dCCA are mostly diagnosed at an earlier stage than iCCA because they cause biliary obstruction when small. iCCA remains often asymptomatic until advanced stages, presenting as a large liver lesion and often with distant metastases.^2^ Early diagnosis of GBC is usually made incidentally at pathological examination after cholecystectomy for stone disease; patients with symptomatic GBC typically have advanced disease.

Consequently, less than 20% of all patients with BTC are eligible for a curative-intent resection or liver transplantation. The median 5-year OS is 20–35%, but recurrence is common, even among patients who survive more than 5 years after resection.^79^^,^^80^ Patients with advanced disease often have a poor performance status (PS) at the time of diagnosis. In a nationwide study, only 13% of all patients with advanced pCCA received palliative systemic treatment due to a combination of tumour advanced stage and poor PS.^81^ Earlier diagnosis, anticipated referral, and appropriate support to improve symptoms and PS would increase the proportion of patients receiving systemic treatment. Inadequate biliary drainage and cholangitis also contribute to clinical deterioration and further decrease the proportion that can receive treatment.^82^^,^^83^

Diagnosis at an earlier stage, however, is challenging, because CCA is rare in Europe, few patients are at increased risk (e.g. PSC), tumour markers have poor sensitivity,^68^ and ultrasound as screening method has limited accuracy. Future studies should identify and validate better biomarkers and imaging modalities for surveillance of high-risk populations. Adequate funding is important to meet this challenge.

The diagnostic work-up of CCA requires an individualised and multimodal approach, based on subtypes and clinical presentation, integrating information derived from CT, MRI including MRCP, and positron emission tomography (PET)-CT and histology.^68^ This work-up may be expensive and time-consuming, leading to a possible delay in final diagnosis and treatment. While ERCP is not performed for surveillance purposes, intraductal sampling for cytology and molecular analyses is recommended in patients with suspicious biliary stenoses, including patients with PSC with relevant strictures, undergoing ERCP.^84^ Interestingly, bile collected during ERCP may also serve as a liquid biopsy matrix. Recent studies support this notion, demonstrating the detection of cancer-associated mutations and epigenetic alterations in cell-free DNA and their clinical significance.^66^^,^^85^^,^^86^ If further validated, these approaches could be a useful complement to current CCA diagnosis and screening methods.

The role of AI for early diagnosis and personalised decision making in patients with CCA is under investigation. AI may offer valid tools to speed up the diagnostic work-up and improve specificity and sensitivity, by combining clinical, biological and radiological features.^87^ Although several studies have been published on this topic, high-quality clinical trials are still needed.

### Need of biomarkers and molecular testing approaches

Recent advances in molecular profiling have improved the knowledge and treatment options of BTC by focusing on gene signatures, single-cell and spatial transcriptomics, and epigenomics. Artificial intelligence (AI), particularly machine learning (ML) to develop statistical algorithms to interpret data, is now being investigated to categorise patients with BTC and make in-depth predictions about their disease course. Various deep learning approaches are emerging and being implemented in pathology and radiological decisions.

Identifying distinct molecular signatures is crucial for categorising patients with BTC and predicting their treatment responses. Using ML, an initial classification of CCA into prognostic subgroups identified gene sets predictive of patient outcomes, especially in aggressive tumours with lymphatic and perineural invasion.^88^ A further detailed characterisation revealed specific targetable genetic alterations, such as *ELF3* mutations and *FGFR2* gene fusions,^89^ with variations in mutation types and frequencies across BTC subtypes suggesting a role for immunomodulatory treatments in hypermutated tumours. An expanded classification incorporating DNA methylation changes identified four distinct tumour clusters with mutations related to carcinogen exposure, suggesting different origins and therapeutic approaches.^90^ The importance of integromics, defining key oncogenic pathways with unique targeted therapeutic options, was also proposed for improved patient stratification.^91^ A gene signature able to distinguish between patients with advanced iCCA who have different chemotherapy outcomes was reported.^92^ A meta-analysis including 1481 iCCAs, mostly surgical specimens, provided a prognostic classification based on clinico-pathological features and tumour molecular profiles. Patients harbouring tumours with *KRAS*, *TP53* and/or *SMAD4* mutations had worse overall survival and lower recurrence-free survival after surgery compared to those with *FGFR2*-fusions, *IDH* mutations, *BAP1* mutations or none of these genetic alterations.^93^ The same study showed differences in stratification between Asian and Caucasian descendants in terms of aetiology, genomics, and histopathology. GBC specific genetic and molecular signatures have been described in relation to the lipid metabolism pathways.^94^ All these data emphasise the importance of molecular profiling in understanding all types of BTC and creating personalised treatment plans.

Advancements in single-cell and spatial transcriptomics are improving our understanding of cellular complexity, interactions, and tissue organisation within BTC. Intratumoural heterogeneity, particularly the presence of osteopontin, has been linked to poor outcomes and therapeutic resistance in liver cancers.^95^ Further investigations identified molecular interactions within the tumour environment correlating with disease severity and patient survival, highlighting specific interactive prognostic markers such as LGALS9-SLC1A5 and SPP1-PTGER4.^96^ These data enforce the need for comprehensive analysis of cell–cell communication and the microenvironment in BTC. A comprehensive understanding of how geographical variations in disease incidence and patient outcomes correlate with differences in the tumour microenvironment remains elusive despite ongoing research into cell–cell communication and the tumour microenvironment within BTCs.

It is interesting to notice that data on the molecular landscape of BTC are often coming from academic centres with high volume of cases, with overrepresentation of American and Asian patients. Impact of various ethnicities on the genetics of BTC is still unexplored, given the lack of representation of all countries. Nonetheless, the increased participation of diversified countries in sponsored clinical trials, and the translational analyses associated with them, will likely fill this gap in the future.

Epigenomic modifications, including DNA methylation and histone modifications, are prevalent and key in the development and progression of CCA.^97^ Four prognostic subgroups of iCCA were identified, with varying degrees of genome disruption and methylation, linked to patient survival.^61^^,^^90^ Additionally, a microRNA signature in blood was reported to distinguish patient with BTC from healthy individuals, with improved diagnostic accuracy when combined with serum CA19-9.^98^ These findings highlight the potential of epigenetic factors in enhancing biomarker discovery and developing new treatment options. However, they still remain exploratory with no clinical applications.

### Overcoming challenges in access to diagnostic testing

Diagnosis of BTCs is often established when the disease is already at an unresectable stage, including locally advanced or metastatic disease, which highly compromises access to effective treatment and results in a dismal outcome.^2^ Early diagnosis currently remains the cornerstone for improving the survival of this devastating disease. The diagnostic tools used to diagnose BTCs range from imaging techniques (e.g. contrast enhanced CT or MRI) to more invasive procedures (e.g. interventional endoscopy, interventional radiology). From a global perspective, it is undebatable that the distribution of human and material resources, including endoscopy or radiology, is highly unbalanced worldwide.^99^^,^^100^ However, even in the industrialised nations, and despite advances in diagnostic modalities, we still face several challenges in both access to comprehensive diagnostic testing and to the required technical expertise often available in only limited number of specialised centres.

Historically, the diagnostics sector has received insufficient awareness in health strategy plans and health expenditure budgets have prioritised access to treatment. As highlighted by the Lancet Commission on diagnostics in 2021, 47% of the global population do not have access to the diagnostic testing essential for six common medical conditions, including diabetes, hypertension, HIV, tuberculosis, and hepatitis B virus infection and syphilis for pregnant women.^101^ Access to the diagnostic tools required in rare cancers, including BTCs, is probably limited to a very small percentage of potential patients. From our survey we observed that all Institutions have access to CT or MR scanning and use these technologies according to the primary site of the tumour. However, we have to acknowledge that all were academic centres and may not reflect the general situation of peripheral and community hospitals.

Molecular profiling has become increasingly relevant in the management of patients with advanced BTCs to enable a precision medicine strategy; guidelines recommend early molecular profiling using Next Generation Sequencing (NGS) for accurate diagnosis and management of BTCs.^68^ Nevertheless, pathologists face several practical challenges during tissue sampling and processing: Obtaining high quality and sufficient tissue samples can be technically challenging, especially from often hard-to-reach anatomical sites. In addition, many BTCs showcase a pronounced tumour stroma, that can dominate the biopsy and poses an additional challenge to the diagnostic workup as well as subsequent analyses, sometimes necessitating repeated biopsy. However, biopsies are associated with patient discomfort, painful procedures, and may delay timely intervention in a frequently medically unfit patient population. Indeed, although there is a general effort to achieve pathological diagnosis in all patients with BTC before starting non-surgical treatments, it is acknowledged that, in at least 20% of centres, priority for initiating therapies is favoured over performing more than two attempts of histological tests, underlining the lack of material, especially for pCCA (Fig. 3). Furthermore, while NGS testing should be offered to all patients who are able to receive treatment, availability and cost of NGS tests might limit its comprehensive use.

Finally, diagnosis of BTC demands specialised MDT and collaboration across disciplines, often challenging traditional frameworks.^71^

## Discussion

Epidemiological data on CCA in Europe reveal significant geographic variations in age-standardised mortality rates, parallel to incidence, with worse outcomes in some Eastern European countries compared to Western countries, but it should be noted that no information is available for some Eastern European countries or for the different types of BTC. It is crucial to determine whether these data reflect the fact that in Western countries there is more awareness of this type of tumour. The ENSCCA Registry, expanded through the EURO-CHOLANGIO-NET and Precision-BTC-Network COST Actions, has proven to be a valuable tool for obtaining information on the CCA landscape in Europe in terms of diagnosis, prognosis and therapeutic insights,^2^^,^^102^^,^^103^ but until recently Eastern European countries were underrepresented in the registry. It is expected that the ENSCCA Registry will in the future include CCA cases from more European countries and also GBC cases and that new studies will provide relevant information on patient characteristics and distribution of these tumours in Europe. Nonetheless, real world data reflect the current clinical practice and mimic the formal coding of the disease. Therefore, it is of paramount importance that a correct coding is introduced and implemented in routine practice to extract solid data on epidemiology and paths to early diagnosis. With this regard the involvement of patients’ charities and governance bodies in a multi-stakeholder effort with physicians is essential, as recently demonstrated by the application of the new ICD-coding system.

The rising incidence of CCA, especially among the younger population, highlights a change in the demographic profile of those affected by this disease and underscores the critical need of early diagnosis and increased awareness among HCPs and the public to improve management of these patients. Decreasing age opens up new social and economic issues for this tumour type, as 30% of patients with BTC are diagnosed before the age of 60, when they are in active employment, which has an impact on the economic performance of a country. Furthermore, the female-specific incidence of GBC and its association with hormonal replacement treatments highlight the importance of addressing sex disparities in cancer prevention and care, as well as in research approaches.^104^

Although in Europe the development of BTC is associated with biliary diseases, metabolic conditions such as obesity and lifestyle factors like tobacco smoking, a significant proportion of cases of BTC are diagnosed without known risk factors, which justifies further research to identify other possible aetiological factors and, in the meantime, awareness should be raised to reduce preventable risk factors.

Imaging modalities and endoscopic techniques are crucial for early diagnosis, but their availability and utilisation vary significantly between regions. There is a notable disparity in access to advanced diagnostic tools and multidisciplinary care, with rural and economically disadvantaged areas often facing greater challenges. This unequal access can delay diagnosis and treatment, worsening patient outcomes.

Molecular testing is revolutionising the understanding and treatment of BTC. Ongoing progress in genomics and epigenomics, combined with advancements in single-cell and spatial analysis, are improving BTC diagnostics and patient care. Deep learning strategies in pathology and radiology for BTC have not yet reached the integration level seen with other genome-wide approaches, but recent studies have demonstrated the applicability of AI in reclassifying combined HCC-CCA with enhanced diagnostic accuracy.^105^^,^^106^ These advancements contribute to more precise and efficient histopathologic classification of liver cancers, including CCA. While practical, ethical and legal constraints are relevant for the widespread implementation of AI in BTC management, these technologies could automate some diagnostic tasks and provide new biomarkers, ultimately improving patient management. The full utility of deep learning in biomarker development will likely be realised through integrating various molecular, imaging and clinical datasets in large patient cohorts. This convergence of technologies is moving towards a future where personalised and precise therapies are commonplace for patients with BTC, which will greatly improve their outcomes.

MDTs play a crucial role in the effective management of patients with BTC, improving diagnostic accuracy and adherence to treatment guidelines. However, access to these specialised care teams and advanced diagnostic tools varies across Europe, influenced by geographic and socioeconomic factors. Not only is important that MDTs are established, but also a harmonised recommendation for the discussion of patients should be pursued to make sure that all patients with BTC receive a multidisciplinary management plan that improves their clinical outcome. This disparity highlights the need for enhanced collaboration and standardisation in diagnostic and treatment protocols to ensure equitable care for all patients with BTC.

## Conclusion

Although there are geographical variations in the epidemiology of BTC in Europe, it is imperative to obtain information from Eastern countries and to obtain more detailed information from all European countries. The increasing incidence of iCCA in Western countries contrasts with stable or declining rates of eCCA and higher rates of CCA in certain regions. In addition, GBC has a higher incidence in Eastern European Countries. Addressing these disparities requires improved awareness and equitable access to advanced diagnostic tools and multidisciplinary care across Europe. Increased collaboration and standardisation of management protocols are essential to improve outcomes for patients with BTC across the continent. Overcoming challenges in access to diagnosis and addressing regional controversies will be key to advancing care and reducing BTC-associated mortality rates.

## Contributors

L.R., R.I.R.M., and C.B. contributed to the conceptualisation, supervision, investigation/data acquisition, visualisation, writing–original draft, writing–review & editing; E.F., S.K., V.C. contributed to the investigation/data acquisition, writing–original draft, writing–review & editing. A.L., A.S., B.G.K., C.G., C.R., G.C., H.M., I.B., J.B.A., J.C., J.-C.N., J.M.B., J.N.K., M.A.A., M.K., and S.R. contributed to writing–original draft, writing–review & editing.

## Editor note

The Lancet Group takes a neutral position with respect to territorial claims in published maps and institutional affiliations.

## Declaration of interests

LR reports grant/research funding (to institution) from AbbVie, Agios, AstraZeneca, BeiGene, Eisai, Exelixis, Fibrogen, Incyte, IPSEN, Jazz Pharmaceuticals, Lilly, MSD, Nerviano Medical Sciences, Roche, Servier, Taiho Oncology, TransThera Sciences, and Zymeworks; consulting fees from AbbVie, AstraZeneca, Basilea, Bayer, Bristol Myers Squibb, Eisai, Elevar Therapeutics, Exelixis, Genenta, Hengrui, Incyte, IPSEN, IQVIA, Jazz Pharmaceuticals, MSD, Nerviano Medical Sciences, Roche, Servier, Taiho Oncology, and Zymeworks; lecture fees from AstraZeneca, Bayer, Bristol Myers Squibb, Guerbet, Incyte, IPSEN, Roche, and Servier; and travel expenses from AstraZeneca. She is chair for the EORTC CITCG HBP/NET Task Force, treasurer for the International Liver Cancer Association, and Special Expert Clinical Trials Europe for NCI GISC Hepatobiliary (HB) Task Force (unpaid positions).

BGK received equipments and materials from Tricumed and Intera Oncology.

JBA received consulting fees from AstraZeneca, and grants (to institution) from Incyte and Adcendo.

AL received consulting fees from Advanz Pharma, GSK, Ipsen, Gilead, Dr Falk, and AstraZeneca; speaker fees from Ipsen, Gore, AstraZeneca, Gilead, Abbvie, Advanz Pharma, AlfaSigma, GSK, and Incyte; support for attending meetings and/or travel from Ipsen; research funding (to institution) from Mirum, GSK, Ipsen, Dr. Falk, Intercept Pharma; and she is the Chair of the Scientific Committee of EASL.

JCN received grants from Ipsen and Bayer, serves on Data Safety Monitoring Board for the Liver-NET1 study (NETRIS pharma), and has four patents [patent N°12306145.9 N° 61/714,383 “A new method for classification of liver samples and diagnosis of focal nodule dysplasia, hepatocellular adenoma, and hepatocellular carcinoma.”; patent N°12306146.7 and N° 61/704,360 “A new method for prognosis of global survival and survival without relapse in hepatocellular carcinoma”; patent B01811/WO-US; Réf. IT: BIO18522 US n° 17/605,524 “New adeno-associated virus (aav) variants and uses thereof for gene therapy”; patent PCT/FR2024/050073 “Methods of predicting the risk of recurrence and/or death of patients suffering from a hepatocellular carcinoma (HCC)”].

JNK reports grant (to institution) from GSK; consulting services for Bioptimus, Owkin, DoMore Diagnostics, Panakeia, AstraZeneca, Mindpeak, and MultiplexDx; lecture fees from AstraZeneca, Bayer, Daiichi Sankyo, Eisai, Janssen, Merck, MSD, BMS, Roche, Pfizer, and Fresenius; and stock options from StratifAI GmbH, Synagen GmbH.

JMB declares consulting/advisory role from Albireo, Ipsen, Cymabay, Ikan Biotech, OWL-Rubió Metabolomic, Jazz Pharmaceuticals, AstraZeneca, and Servier; honoraria/lectures from Incyte, AstraZeneca, Intercept, Eisai, and Advanz), and research funding from Albireo, Incyte and Cymabay.

IB reports consulting fees from Sirtex, Terumo, Boston Scientific, Roche, AstraZeneca, Eisai, Microbot Medical; lecture fees from AstraZeneca, Boston Scientific, Eisai, GE Healthcare, Guerbet, Roche, Sirtex, Terumo; travel expenses from Boston Scientific, Sirtex; and serves on Independent Data Safety Monitoring Board for AstraZeneca.

AS received honoraria as speaker from BMS, Roche, Servier, Ipsen, Lilly, AstraZeneca, MSD, Eisai, travel support from Ipsen, Servier, Pierre-Fabre, MSD, Eisai, and declares advisory role for Heparegenix.

VC reports grants from Ipsen, speaking fees from Ipsen, travel expenses from Advanz, and advisory role for Ipsen.

CB received honoraria as speaker (Astrazeneca, Incyte, Servier) and consultant (Incyte, Servier, Boehringer Ingelheim, Astrazeneca, Tahio, Jazz Pharmaceuticals, Molecular Partners), received research funds (Avacta, Medannex, Servier) and her spouse is an employee of Astrazeneca.

RIRM reports institutional funds from AstraZeneca, Incyte, Servier, Taiho and Jazz Pharmaceuticals.

All other authors declare no competing interests.
