# Supplementary material for: Mapping the landscape of biliary tract cancer in Europe: challenges and controversies
Source: Lancet Reg Health Eur. 2025 Feb 19;50:101171. doi: 10.1016/j.lanepe.2024.101171 (PMC11910794; doi:10.1016/j.lanepe.2024.101171)
Supplement: Supplementary Table S1 [file mmc1.docx]

**Supplementary Material**

**Mapping the landscape of biliary tract cancer in Europe: Challenges and controversies**

**Supplementary File 1**. **Survey of Clinical practice in BTC in Europe carried out using google forms.**

| **Question** | **Options for participants** |
| --- | --- |
| Please, indicate your clinical specialty |  |
| Type of centre | Academic  Community |
| Your location - city/town |  |
| Your location - country |  |
| Do you apply international (ESMO/EASL) guidelines in clinical practice? | Yes  No |
| At your centre, do you have dedicated oncologists? | Yes  No |
| At your centre, do you have dedicated gastroenterologists? | Yes  No |
| At your centre, do you have dedicated surgeons? (including consideration of liver transplantation) | Yes  No |
| At your centre, do you have a dedicated tumour board? | Yes  No  Other, please specify |
| Do you discuss all patients with BTC at a dedicated tumour board? | Only at diagnosis  At the time of each therapeutic decision  Only patients candidate to surgery or locoregional therapies  All patients at diagnosis and occasionally during the treatment pathway if feedback from gastroenterologists, radiologists or surgeons is needed |
| If you don’t discuss all patients at the dedicated tumour board, which percentage of them do you discuss? | <25%  25-50%  51-75%  >75%  Other, please specify |
| Do you always proceed with pathological diagnosis before any non-surgical treatment? | Yes  No  Other, please specify |
| Do you ask for CA 19-9 at diagnosis and the time of tumour assessments? | Yes  No  Other, please specify |
| Do you ask for CEA and AFP at diagnosis and at the time of tumour assessments? | Yes  No  Other, please specify |
| Which imaging procedures do you use for staging and tumour assessments? | Thoraco-abdomino-pelvic CT  MRI / MRCP  18FDG-PET scan  CT and MRI  CT, MRI and PET (according to patients’ characteristics)  CT (MRI and PET only in selected cases) |
| Do you always carry out radiological imaging and MDT discussion before ERCP or PTC and drainage in patients with jaundice? | Yes  No  Other, please specify |
| Please add any comments regarding your clinical practice that you feel are relevant to you and that have not been reflected in the questions above |  |
